# Association between anti-*Porphyromonas gingivalis* or anti-α-enolase antibody and severity of periodontitis or rheumatoid arthritis (RA) disease activity in RA

**DOI:** 10.1186/s12891-015-0647-6

**Published:** 2015-08-12

**Authors:** Joo Youn Lee, In Ah Choi, Jin-Hee Kim, Kyoung-Hwa Kim, Eun Young Lee, Eun Bong Lee, Yong-Moo Lee, Yeong Wook Song

**Affiliations:** 1 Department of Molecular Medicine and Biopharmaceutical Sciences, Graduate School of Convergence Science and Technology and College of Medicine, Medical Research Center, Seoul National University, Seoul, South Korea; 2 Division of Rheumatology, Department of Internal Medicine, College of Medicine, Seoul National University, Seoul, South Korea; 3 Department of Periodontology, School of Dentistry, Seoul National University, Seoul, South Korea; 4 Dental Research Institute, School of Dentistry, Seoul National University, Seoul, South Korea

## Abstract

**Background:**

Periodontitis (PD) has been reported to be associated with rheumatoid arthritis (RA). *Porphyromonas gingivalis* (*P. gingivalis*) is a gram-negative anaerobic bacterium that is recognized as one of the major pathogenic organisms in PD and is the only bacterium known to express peptidylarginine deiminase (PAD). Antibody against human α-enolase (ENO1) is one of the autoantibodies in RA. ENO1 is a highly conserved protein, and could be a candidate molecule for molecular mimicry between bacterial and human proteins. In the present study, we measured serum antibody against *P. gingivalis* and human ENO1 in patients with RA and investigated their association with the severity of PD or disease activity of RA.

**Methods:**

Two hundred, forty-eight patients with RA and 85 age- and sex-matched healthy controls were evaluated by rheumatologic and periodontal examinations. The serum levels of anti-*P. gingivalis* and anti-ENO1 antibodies were measured by an enzyme-linked immunosorbent assay (ELISA).

**Results:**

Patients with RA had significantly higher levels of anti-*P. gingivalis* and anti-ENO1 antibody titers than the controls (*p* = 0.002 and 0.0001, respectively). Anti-*P. gingivalis* antibody titers significantly correlated with anti-ENO1 antibody titers in RA patients (r = 0.30, *p* < 0.0001). There were significant correlations between anti-*P. gingivalis* antibody titers and the gingival index (GI), probing pocket depth (PPD), bleeding on probing (BOP) and clinical attachment level (CAL) (*p* = 0.038, 0.004, 0.004 and 0.002, respectively) in RA. Anti-*P. gingivalis* antibody titers were not correlated with disease activity score 28 (DAS28) or anti-CCP titer. However, anti-ENO1 antibody titers were significantly correlated not only with the periodontal indices, such as PPD, BOP, and CAL (*p* = 0.013, 0.023 and 0.017, respectively), but also RA clinical characteristics, such as DAS28, anti-CCP titer, and ESR (*p* = 0.009, 0.015 and 0.001, respectively).

**Conclusion:**

Anti-*P. gingivalis* and anti-ENO1 antibody titers were correlated with the severity of PD in RA. Anti-ENO1 antibody titers, but not anti-*P. gingivalis* antibody titers, were further associated with RA disease activity.

**Electronic supplementary material:**

The online version of this article (doi:10.1186/s12891-015-0647-6) contains supplementary material, which is available to authorized users.

## Background

Rheumatoid arthritis (RA) is chronic inflammatory autoimmune disease characterized by persistent synovitis, systemic inflammation, production of autoantibodies, and bone destruction of joints. RA is more frequent among women than men (3:1) and its prevalence is 0.5-1.0 % in the adult population [1–3]. The etiology of RA remains unknown, but genetic (including HLA-DRB1) and environmental factors, such as smoking [4–6] and infection [7–9], play a considerable role in disease susceptibility [10]. Periodontitis (PD) is one of the most common chronic disorders of infectious origin with a prevalence of 10-60 % in adults [11]. PD is caused by a chronic infection of twenty different bacterial species, of which *Porphyromonas gingivalis* (*P. gingivalis*) is the most common. A large number of clinical studies have shown an increased frequency of PD in patients with RA as compared to individuals without RA [12–15]. One report indicated that the incidence of RA in patients with PD is a 3.95 % compared to a 1 % prevalence in the general population [16]. RA and PD share several genetic risk factors, such as HLA-DR4-subtypes 0401, 0404, 0405, 0408 [17] and environmental factors such as smoking [18, 19], and both diseases are characterized by chronic self-sustaining inflammation [20]. These results suggest that there could be a positive association of RA with PD, an infectious disease initiated by oral anaerobic bacteria. It has been hypothesized that this association may be based on the capacity of *P. gingivalis*, the major etiological agent of PD, which express a peptidylarginine deiminase (PAD), an enzyme that catalyzes the transformation of arginine to citrulline [21]. *P. gingivalis* PADs (PPAD) are capable of citrullinating an endogenous or human protein [22], thereby creating systemic immunogens that contain epitopes against which anti-citrullinated protein antibodies (ACPAs) could be raised [21]. The correlation between anti-*P. gingivalis* titers and periodontal indices, such as PPD and CAL, has been reported in previous studies [23–25]. In contrast, no correlation has been shown between anti-*P. gingivalis* and RA disease activity score 28 (DAS28) [25].

The antibody against human α-enolase (ENO1) is the autoantibody reported in 6-66 % of RA patients [26–29]. ENO1 is a highly conserved protein and could be a candidate for molecular mimicry between bacterial and human host proteins [30]. There is evidence of homology and cross-reactivity between the enolase of *P. gingivalis* and its human origin [9, 31, 32]. Endogenous citrullinated enolases have been reported to be abundant in *P. gingivalis* [33]. There has been no report on the association of anti-ENO1 and periodontitis or RA disease activity. In this study, we investigated serum antibody responses to *P. gingivalis* and human ENO1 in patients with RA compared to controls. Then, we examined whether anti-*P. gingivalis* and anti-ENO1 antibodies are associated with the periodontal indices and RA disease activity.

## Methods

### Study population

This study was approved by the ethics committee of Seoul National University Hospital. RA patients (*n* = 248) were enrolled at the Rheumatology Clinic at the Seoul National University Hospital from May 2011 to February 2012 and satisfied the 1987 American College of Rheumatology classification criteria of RA [34]. The non-arthritic controls (*n* = 85) were age- and sex-matched volunteers in a 3:1 ratio. The institutional review board and ethics committee approved the protocol (H-1103-151-357), and written informed consent was obtained from each patient before study enrollment.

### Clinical assessment

We conducted a prospective, cross-sectional study comparing RA patients and non-arthritic controls. Patients underwent interviews to determine socio-demographic data, medical history, and comorbidities. In RA patients, clinical parameters including 68 tender joint count (TJC), 66 swollen joint count (SJC), and the patient’s global assessment of disease activity on a visual analogue scale of 100 mm was evaluated. Joint count was performed by a single rheumatologist (IAC) to minimize interobserver variability. Disease activity score 28 (DAS28) was calculated as follows [35, 36]; $\left[0.56\times \sqrt{\left(28\kern0.5em \mathrm{tender}\ \mathrm{joint}\ \mathrm{count}\right)} + 0.28\times \sqrt{\left(28\ \mathrm{swollen}\ \mathrm{joint}\ \mathrm{count}\right)}+\kern0.2em 0.70\times \mathrm{L}\mathrm{n}\left(\mathrm{erythrocyte}\ \mathrm{sedimentation}\ \mathrm{rate}\right)\right]\times 1.08+0.16$.

RA disease duration, morning stiffness, erythrocyte sedimentation rate (ESR), rheumatoid factor (RF), anti-cyclic citrullinated peptide (CCP) antibody, and the presence of erosive changes on X-rays of joints were evaluated when serum samples were obtained.

In all subjects, the number of teeth (0 - 28, 3rd molars excluded) was checked. Subjects who had 15 or more teeth were evaluated with a dental exam and checked for anti-*P. gingivalis* and anti-ENO1 antibodies. Two periodontologists (JK and YMK) who had been trained for the periodontal index for more than two years in the same clinic performed dental exam. The plaque index (PI) was used as a marker of dental hygiene and graded as 1, 2, and 3 at three buccal points and one lingual point in each tooth [37]. Mean values of a maximum of 112 points were used, and higher PI represented poorer dental hygiene. Gingival index (GI), probing pocket depth (PPD), bleeding on probing (BOP), and clinical attachment level (CAL) were evaluated as indices of periodontitis. GI was graded as 1, 2, and 3 at three buccal points and one lingual point in each tooth. Mean values of a maximum of 112 points were used and higher index represented greater gingival inflammation [38]. PPD was measured by a 15 mm-University of North Carolina (UNC) probe in mm scale and higher index scores represented more severe structural changes; values over 4 mm were considered to be a pathologic condition. BOP was checked as positive/negative, coded as 1/0, and positive BOP represented an early sign of inflammation. The mean value of each tooth was presented in percentage. CAL was taken as the distance from the cementoenamel junction (CEJ) to the base of the probable crevice. It was calculated as sum of PPD and gingival recession measured with the 15 mm-UNC probe in mm scale. It was regarded as a practical index of periodontitis. Periodontitis was further defined as slight (CAL 1–2 mm), moderate (CAL 3–4 mm), and severe (CAL ≥5 mm) according to American Academy of Periodontology 2004 classification [39]. PPD, BOP, and CAL were checked at three buccal points and three lingual points in each tooth and the mean value of a possible maximum 168 points was used.

### Antibody to *P. gingivalis*

The *P. gingivalis* strain *FDC381* was grown in brain heart infusion (BHI) broth (Difco, Detroit, MI) supplemented with hemin (5 μg/ml), vitamin K (0.5 μg/ml), and cysteine (0.05 %). Cultures were performed under anaerobic conditions (GasPak-EZ anaerobe container system, Becton Dickinson Microbiology systems, MD, USA) at 37 °C for 3 days. After removal of the media, the cells were washed with phosphate-buffered saline (PBS) three times, and then treated with 3 % formaldehyde as a fixative. After centrifugation, *P. gingivalis* cells were washed with 50 mM sodium carbonate coating buffer (pH 9.6) and then the number of *P. gingivalis* cells was determined by spectrophotometer.

Each well of the 96-well microtiter plate (NUNC, Roskilde, Denmark) was coated with 1x10^7^ cells/well of *P. gingivalis* cells in 50 mM sodium carbonate coating buffer (pH 9.6) overnight at 4 °C. After washing three times with PBS containing 0.05 % Tween20 (PBST, pH 7.4) and blocking with PBS containing 2 % bovine serum albumin (BSA), two-fold serial dilutions of RA patients and control sera (first dilution, 1:200) were added to the plate and the bound human IgG was detected with HRP-conjugated, anti-human IgG antibodies (Millipore, Billerica, MA, USA, 1/6,000 dilution), followed by a developer containing TMB (KPL, Gaithersburg, MD). The anti-*P. gingivalis* titer was defined as the inverse value of the largest serial dilution for which detectable antibody was observed.

### Antibody to ENO1

Each well of the 96-well microtiter plate (NUNC, Roskilde, Denmark) was coated with 1 μg/ml of human ENO1 recombinant protein (Prospec, Ness-Ziona, Israel) in 50 mM sodium carbonate coating buffer (pH 9.6) overnight at 4 °C. After washing three times with PBST, and blocking with PBS containing 2 % BSA, two-fold serial dilutions of RA patients and control sera (first dilution 1:200) were added to the plate and the bound human IgG was detected with HRP-conjugated, anti-human IgG antibodies (Millipore, 1/6,000 dilution) followed by a developer containing TMB (KPL, Gaithersburg, MD). The anti-ENO1 titer was defined as the inverse value of the largest serial dilution for which detectable antibody was observed.

### Serum RF and anti-CCP antibody

The values of serum RF were measured by the immunoturbidimetry method (Roche, Swiss), and anti-CCP antibody titer was measured by chemiluminescent microparticle immunoassay (Abbott, USA) according to the manufacturer’s instructions. Anti-CCP antibody titer over 5 international unit (IU)/mL was considered as positive.

### Statistical analyses

Differences in demographic and clinical parameters were assessed by Mann–Whitney U tests for the comparison of continuous variables, and chi-square or Fisher’s exact test for categorical variables. Serum levels of antibody to *P. gingivalis* and ENO1 between the RA and control groups were compared by non-parametric Mann–Whitney U tests.

The correlations between serum antibody to *P. gingivalis* or ENO1 with RA clinical characteristics or periodontal indices were examined by determining Spearman correlation coefficients, as appropriate. Multiple logistic regression models were used to compare titers of antibodies to *P. gingivalis* and ENO1 between RA patients and controls, adjusting for age, sex, and smoking status. All reported *p* values were two-sided and *p* <0.05 was considered to indicate statistical significance. Analysis was performed by using IBM SPSS 19.0 and GraphPad Prism 5.

## Results

Demographic and periodontal characteristics of the study participants are summarized in Table 1. There were no significant differences in age, sex, smoking status, and the number of remaining teeth between the RA and control groups. However, RA patients had higher levels of clinical periodontal indices such as PI (mean ± SE, 0.85 ± 0.03 vs. 0.69 ± 0.03, *p* = 0.014 by Mann–Whitney test), GI (0.51 ± 0.03 vs. 0.14 ± 0.02, *p* < 0.0001), PPD (20.42 ± 0.98 vs. 11.69 ± 1.00, *p* < 0.0001), BOP (1.97 ± 0.02 vs. 1.74 ± 0.02, *p* < 0.0001), and CAL (3.25 ± 0.05 vs. 2.89 ± 0.05, *p* < 0.0001) compared to the controls (Table 1). A higher prevalence of moderate and severe PD was observed in RA patients compared with control subjects (*p* < 0.0001 by chi-square test; slight vs. moderate to severe).

**Table 1:** Demographic and periodontal characteristics of patients with RA and healthy controls

Parameter	RA	Healthy controls	*p* value
	(*n* = 248)	(*n* = 85)	
Age (years, mean ± SE)	60.1 ± 0.7	59.13 ± 1.3	0.512
Female (n, %)	218 (87.9)	74 (87.1)	0.995
Smoking status^a^			
Current (%)	5 (2.1)	4 (4.7)	0.241
Former (%)	9 (3.8)	4 (4.7)	0.746
Never (%)	224 (94.1)	77 (90.6)	0.228
Severity of periodontitis			
Slight (%)	88 (35.5)	57 (67.1)	<0.0001^†^
Moderate (%)	153 (61.7)	28 (32.9)	
Severe (%)	7 (2.8)	0 (0)	
Number of remaining teeth (mean ± SE)	25.3 ± 0.2	25.9 ± 0.3	0.063
Periodontal indices			
Plaque index (PI)	0.85 ± 0.03	0.69 ± 0.03	0.014
Gingival index (GI)	0.51 ± 0.03	0.14 ± 0.02	<0.0001
Probing pocket depth (PPD)	20.42 ± 0.98	11.69 ± 1.00	<0.0001
Bleeding on probing (BOP)	1.97 ± 0.02	1.74 ± 0.02	<0.0001
Clinical attachment level (CAL)	3.25 ± 0.05	2.89 ± 0.05	<0.0001

RA, rheumatoid arthritis; SE, standard error; slight periodontitis, clinical attachment loss 1–2 mm; moderate periodontitis, clinical attachment loss 3–4 mm; severe periodontitis, clinical attachment loss ≥ 5 mm by the American Academy of Periodontology 2004 classification

^a^Smoking status of 10 RA patients could not be obtained

^†^*p* value by chi-square test (slight vs. moderate and severe)

**Fig. 1:**
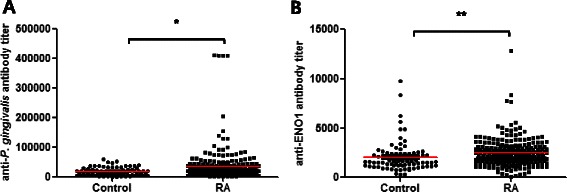
Serum antibody titers to *Porphyromonas gingivalis* (**a**) and α-enolase (**b**) in RA patients and healthy controls. * *p* < 0.003, ** *p* < 0.0001 by Mann-Whitney U-test; bars represent mean. *P. gingivalis*, *Porphyromonas gingivalis*; ENO1, α-enolase

**Fig. 2:**
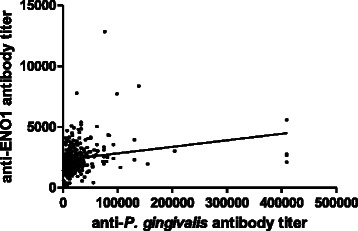
Correlation between anti-*P. gingivalis* and anti-ENO1 antibody titers in RA patients. r = 0.30, *p* < 0.0001 by Spearman test. *P. gingivalis*, *Porphyromonas gingivalis*; ENO1, α-enolase

The serum antibody titers to *P. gingivalis* (mean ± SE, 34,427 ± 3,510 vs. 18,479 ± 1,428, *p* = 0.003 by Mann–Whitney test) and ENO1 (2,473 ± 87.97 vs. 2,072 ± 167.4, *p* < 0.0001) were significantly higher in patients with RA compared to control subjects (Fig. 1). The differences in anti-*P. gingivalis* or anti-ENO1 antibody titers between patients with RA and control subjects remained statistically significant after adjustments for age, sex and smoking status (*p* = 0.002 and 0.032, respectively). Anti-*P. gingivalis* antibody titers significantly correlated with anti-ENO1 antibody titers in RA patients (r = 0.30, *p* < 0.0001 by Spearman test, Fig. 2).

We analyzed the association between anti-*P. gingivalis* or anti-ENO1 antibody titers and periodontal indices or RA clinical characteristics. Anti-*P. gingivalis* antibody titers correlated with values of periodontal indices such as GI (r = 0.13, *p* = 0.038 by Spearman test), PPD (r = 0.18, *p* = 0.004), BOP (r = 0.18, *p* = 0.004), and CAL (r = 0.19, *p* = 0.002) in RA (Table 2). However, anti-*P. gingivalis* antibody titers did not significantly correlate with RA clinical characteristics including DAS28, RF and anti-CCP titer but correlated with ESR (r = 0.16, *p* = 0.011 by Spearman test, Table 3).

**Table 2:** Correlation between titers of anti-*P. gingivalis* or anti-ENO1 antibody and periodontal indices in patients with RA and controls

Antibody	Group		PI	GI	PPD	BOP	CAL
Anti-*P. gingivalis*	RA	r	0.05	0.13	0.18	0.18	0.19
		*p* value	0.423	0.038	0.004	0.004	0.002
	Control	r	−0.10	−0.02	−0.07	0.04	−0.06
		*p* value	0.360	0.845	0.760	0.741	0.610
Anti-ENO1	RA	r	0.10	0.06	0.16	0.14	0.15
		*p* value	0.106	0.361	0.013	0.023	0.017
	Control	r	0.05	−0.04	−0.03	−0.001	−0.12
		*p* value	0.676	0.724	0.757	0.992	0.279

*P. gingivalis Porphyromonas gingivalis*, *ENO1* α-enolase, *PI* plaque index, *GI* gingival index, *PPD* probing pocket depth, *BOP* bleeding on probing, *CAL* clinical attachment level

**Table 3:** Correlation between titers of anti-*P. gingivalis* or anti-ENO1 antibody and clinical characteristics of RA

Antibody		DAS28	Duration of morning stiffness	ESR	RF titer	Anti-CCP titer
Anti-*P. gingivalis*	r	0.10	−0.01	0.16	0.08	0.08
	*p* value	0.143	0.937	0.011	0.216	0.250
Anti-ENO1	r	0.17	0.02	0.21	0.13	0.17
	*p* value	0.009	0.793	0.001	0.052	0.015

*P. gingivalis Porphyromonas gingivalis*, *ENO1* α-enolase, *DAS28* disease activity score 28, *ESR* erythrocyte sedimentation rate, *RF* rheumatoid factor, *CCP* cyclic citrullinated peptide

Serum anti-ENO1 antibody titers showed statistically significant correlations with values of PD indices such as PPD (r = 0.16, *p* = 0.013 by Spearman test), BOP (r = 0.14, *p* = 0.023), and CAL (r = 0.15, *p* = 0.017) in RA (Table 2). In addition, anti-ENO1 antibody titers correlated with RA clinical characteristics such as DAS28 (r = 0.17, *p* = 0.009 by Spearman test), ESR (r = 0.21, *p* = 0.001), and anti-CCP antibody titer (r = 0.17, *p* = 0.015) in RA patients (Table 3). There was no correlation of anti-*P. gingivalis* or anti-ENO1 and periodontal indices in the healthy controls.

## Discussion

The association between RA and PD has been reported in clinical studies [12–15, 40, 41] and a population-based study [42]. A large number of clinical studies have shown an increased frequency of PD in patients with RA as compared to individuals without RA. Also in our study population, a higher prevalence of moderate and severe PD was observed in RA patients compared with non-RA control subjects. We showed significantly elevated antibody responses to *P. gingivalis* and ENO1 in RA patients compared to controls. These findings are consistent with the results of previous reports on anti-*P. gingivalis* [43, 44] and anti-ENO1 [26, 27]. Anti-*P. gingivalis* antibody titers significantly correlated with anti-ENO1 antibody titers in RA patients. To exclude the confounding effect by PD status, we subdivided subjects into slight PD and moderate PD depending on the severity of PD. Serum anti-*P. gingivalis* antibody titer was not different between slight PD and moderate PD subgroup in non-RA controls (*p* = 0.8736). Similarly, anti-ENO1 antibody titer was not different between slight PD and moderate PD subgroup in non-RA controls (*p* = 0.2578). But in RA patients group, serum anti-*P. gingivalis* (*p* = 0.039) and anti-ENO1 (*p* = 0.0147) antibody titer were higher in moderate PD subgroup than slight PD subgroup (Additional file 1: Figure S1). In RA patients, anti-*P. gingivalis* antibody titers correlated with periodontal destruction represented as PPD and CAL as described in previous studies [23–25]. Moreover, in the present study the anti-*P. gingivalis* antibody titer correlated with gingival inflammation indices, such as GI and BOP as well. However, anti-*P. gingivalis* antibody titers did not correlate with RA disease activity. Previous studies have shown that antibodies to *P. gingivalis* are associated with ACPAs, such as anti-CCP antibody in patients with RA [45, 46]. However, antibodies against *P. gingivalis* in seropositive arthralgia patients were not shown to predict the development of rheumatoid arthritis [45]. In our cohort, titers of anti-*P. gingivalis* antibody were not correlated with the titers of anti-CCP antibodies.

Anti-ENO1 antibody titers showed a similarly significant correlation with PPD, BOP, (reflecting gingival inflammation) and CAL (periodontal destruction) in RA patients. In addition, anti-ENO1 antibody titers correlated with RA disease activities, such as DAS28, ESR, and anti-CCP titer (*p* = 0.009, 0.001, and 0.015, respectively) in RA patients. Fisher *et al*. reported that there were trends towards higher C-reactive protein (CRP), DAS28 (CRP) and also greater use of methotrexate in the anti-citrullinated α-enolase peptide 1 (CEP-1)+/CCP2+ subset than in the anti-CEP-1-/CCP2+ subset in one cohort (Norfolk Arthritis Register cohort, *p* = 0.08) [47]. We measured the serum antibody against whole ENO1 protein, but not against citrullinated peptide. The correlation with anti-ENO1 and anti-CCP antibody titers suggests that patients with anti-ENO1 antibodies might have antibodies against citrullinated ENO1 due to epitope spreading. Anti-ENO1 antibody titers were significantly higher in RA patients with anti-CCP antibodies than patients without anti-CCP antibodies (*p* = 0.037 by Mann–Whitney test, data not shown). Evidence of cross-reactive immune responses *in vivo* was previously reported in an animal model, in which immunization of DR4 transgenic mice with *P. gingivalis* enolase, both the citrullinated and uncitrullinated forms, caused rapid-onset arthritis [32]. *P. gingivalis* enolase and human ENO1 share 51.4 % amino-acid homology, and 82 % homology at the 17-amino acid immunodominant regions [9, 32]. It was suggested that *P. gingivalis* may have a role in breaking tolerance to human ENO1 [9, 31, 32]. Therefore, elevated anti-ENO1 titers in RA may be due to a cross-reactive immune response to *P. gingivalis* enolase. Antibodies against ENO1 have been found in a variety of autoimmune and inflammatory diseases, including RA (6-66 %), systemic lupus erythematosus (19-80 %), mixed cryoglobulinemia (32-64 %), systemic sclerosis (15-30 %), anti-neutrophil cytoplasmic antibody (ANCA)-positive vasculitides (37 %), Behcet’s disease (38-45 %), autoimmune hepatitis (32-56 %), inflammatory bowel disease (10-18 %), and Hashimoto’s thyroiditis (6-83 %) [46]. Anti-ENO1 antibodies were reported to contribute to the perpetuation of synovial inflammation in RA by stimulating monocytes and macrophages to produce increased amounts of proinflammatory mediators, such as TNF-α, IL-1α/β, IFN-γ, and PGE2 via the p38 mitogen activated protein kinase and NF-kB pathways [48]. Our result, that anti-ENO1 is correlated with disease activity, is consistent with those findings.

There are several limitations in our study. Patients with less than 15 teeth or ongoing dental treatment were excluded in the evaluation of periodontitis in order to calculate exact periodontal indices. Therefore, patients with most severe periodontitis might be excluded in our analysis.

Study population in this study has Asian genetic backgrounds, limiting generalizability of our results. However, this population also has a unique strength that most subjects in both RA and control group was a never-smoker. Smoking is a powerful environmental factor for RA and is known to induce PAD secretion with inflammatory conditions in the lungs, contributing to the breakdown of immune tolerance to citrullinated epitopes [4–6]. It is also a major risk factor for PD [18, 19], suggesting a common pathogenic mechanism links the two diseases and being expected to be a major confounder of the study investigating the relation between PD with RA. Low smoking rate of our study population may be appropriate to explain substantial number of non-smokers still developing rheumatoid arthritis and supports the role of *P. gingivalis* and anti-ENO1 in RA, not necessarily linked to smoking. According to the fifth Korea National Health and Nutrition Examination Survey (KNHANES V-3), current smokers in Korean female, stratified by age were 7.9 % in their fifties, and 1.6 % in their sixties, not irrelevant from the results of this study [49].

We also did not match the proportion of smokers when recruiting non-arthritis controls and this could influence the severity of periodontitis. However, there was no statistically significant difference in smoking status between the RA and control groups.

We evaluated anti-*P. gingivalis* antibody and anti-ENO1 antibody in RA with PD. We showed that anti-*P. gingivalis* was associated with the severity of PD in RA but not with RA disease activities or titers of anti-CCP antibody. Anti-ENO1 antibodies were correlated with severity of PD and disease activities in RA.

## Conclusion

Anti-*P. gingivalis* antibodies and anti-ENO1 antibodies were higher in RA patients than in controls. Anti-*P. gingivalis* antibodies correlated with PD parameters in RA patients, but not with RA disease activity. Anti-ENO1 antibodies correlated with not only the periodontal indices but also RA disease activity in RA patients.

## Additional file

Additional file 1: Figure S1. Titers of anti-*P. gingivalis* (A) and anti-ENO1 antibody (B) according to PD severity in healthy control (HC) and RA patients (Figure not shown in the manuscript). (PDF 210 kb)
