# Supplementary material for: Association between anti-Porphyromonas gingivalis or anti-α-enolase antibody and severity of periodontitis or rheumatoid arthritis (RA) disease activity in RA
Source: BMC Musculoskelet Disord. 2015 Aug 12;16:190. doi: 10.1186/s12891-015-0647-6 (PMC4542108; doi:10.1186/s12891-015-0647-6)
Supplement: Additional file 1: Figure S1. — Titers of anti-P. gingivalis (A) and anti-ENO1 antibody (B) according to PD severity in healthy control (HC) and RA patients (Figure not shown in the manuscript). (PDF 210 kb) [file 12891_2015_647_MOESM1_ESM.pdf]

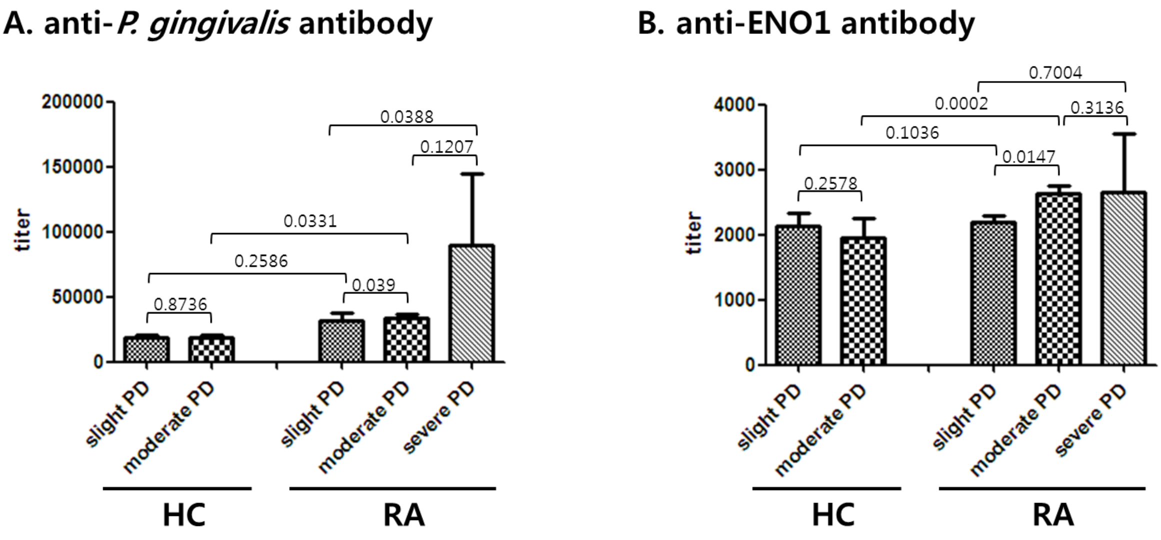

Supplementary figure S1. Titers of anti-*P. gingivalis* (A) and anti-ENO1 antibody (B) according to PD severity in healthy control (HC) and RA patients (Figure not shown in the manuscript).
